# Examining TikTok’s Potential for Community-Engaged Digital Knowledge Mobilization With Equity-Seeking Groups

**DOI:** 10.2196/30315

**Published:** 2021-12-09

**Authors:** Kinnon Ross MacKinnon, Hannah Kia, Ashley Lacombe-Duncan

**Affiliations:** 1 School of Social Work York University Toronto, ON Canada; 2 School of Social Work The University of British Columbia Vancouver, BC Canada; 3 School of Social Work University of Michigan Ann Arbor, MI United States

**Keywords:** trans, nonbinary, marginalized communities, gender-affirming care, digital health, community-engaged research, knowledge mobilization, mobile phone

## Abstract

Social media is increasingly being leveraged by researchers to engage in public debates and rapidly disseminate research results to health care providers, health care users, policy makers, educators, and the general public. This paper contributes to the growing literature on the use of social media for digital knowledge mobilization, drawing particular attention to TikTok and its unique potential for collaborative knowledge mobilization with underserved communities who experience barriers to health care and health inequities (eg, equity-seeking groups). Setting the TikTok platform apart from other social media are the unique audiovisual video editing tools, together with an impactful algorithm, that make knowledge dissemination and exchange with large global audiences possible. As an example, we will discuss digital knowledge mobilization with trans and nonbinary (trans) communities, a population that experiences barriers to health care and is engaged in significant peer-to-peer health information sharing on the web. To demonstrate, analytics data from 13 selected TikTok videos on the topic of research on gender-affirming medicine (eg, hormonal therapy and surgeries) are presented to illustrate how knowledge is disseminated within the trans community via TikTok. Considerations for researchers planning to use TikTok for digital knowledge mobilization and other related community engagement with equity-seeking groups are also discussed. These include the limitations of TikTok analytics data for measuring knowledge mobilization, population-specific concerns related to community safety on social media, the spread of disinformation, barriers to internet access, and commercialization and intellectual property issues. This paper concludes that TikTok is an innovative social media platform that presents possibilities for achieving transformative, community-engaged knowledge mobilization among researchers, underserved health care users, and their health care providers, all of whom are necessary to achieve better health care and population health outcomes.

## Introduction

### Background

Social media is increasingly being leveraged by researchers to engage in scholarly debates and rapidly disseminate research results. Rapid dissemination strategies can facilitate the uptake of life-saving information, fill gaps in critical information, and correct misinformation, as has been documented throughout the COVID-19 pandemic [1]. However, social media also provides a platform for challenging disseminated knowledge that is produced about communities without appropriate community engagement and critical attention to ethical considerations. As an example, significant controversy erupted in 2019 when a researcher published a controversial study that presented a medically unsubstantiated term, *rapid-onset gender dysphoria*, arguing that trans and nonbinary (trans) identity is a *social and peer contagion* [2]. In response, several trans-identified community-engaged researchers challenged these findings on Twitter to draw attention to the study’s methodological flaws and its potential harm to trans-people [3-5]. Scientific critiques of this study were later published by researchers in peer-reviewed journals and again shared through social media and web-based news media channels, leading to corrections made to the original article [6,7]. This is only one striking example demonstrating the transformative potential of community-engaged digital advocacy.

This paper extends the existing research on the use of social media for knowledge mobilization and collaborative knowledge exchange, focusing explicitly on TikTok. It is among the first academic papers to draw attention to this fast-growing social media platform and its potential use as a digital knowledge mobilization tool. In so doing, the article highlights (1) how TikTok functions, including its unique algorithm, which creates the opportunity to reach larger international audiences in comparison with other social media platforms and relatedly, (2) TikTok’s potential for audiovisual knowledge exchange with communities who are active TikTok users—also referred to as *creators* on the platform. As an example, we specifically highlight the possibilities for knowledge mobilization with trans communities, drawing from the first author’s (KRM) TikTok analytics data. Some of TikTok’s anticipated pitfalls and limitations have also been discussed to inform researchers. This viewpoint study is also of practical use to researchers engaging with an expansive number of web-based communities on TikTok, such as youth, and more [8]. Although important, in-depth considerations of the intersections among social media technologies, ethics, and society are out of scope. For interested readers, these discussions may be sought out within science and technology studies and other interdisciplinary literature [9-11].

### Knowledge Mobilization and Health Equity

Knowledge mobilization is of primary concern to researchers whose study findings have tremendous consequences on the health equity of underserved, minoritized populations, who are also termed *equity-seeking groups*. For example, trans individuals constitute a growing population whose shared experiences and health care needs have garnered significant attention in recent years. Trans persons identify with a different gender than was assigned at birth, with many undergoing a gender transition to align their physical characteristics with their internally felt gender identity [12]. Gender transitions may include the use of a new name and gender pronoun and/or changes to one’s physical appearance (eg, clothing and hairstyle); updating some or all of one’s legal identity documentation (eg, passport and driver’s license); or accessing gender-affirming hormonal therapy or surgical procedures, among other social, legal, and medical processes [12]. Although not all trans people seek gender-affirming medicine (eg, hormones and surgeries) to transition, improving access to these medical treatments is of great concern to trans communities and their health care providers. Gender-affirming medicine is associated with improvements in trans people’s mental health outcomes [13,14], and in some jurisdictions, these treatments are a prerequisite for changing legal sex designations [15]. Recently, in the United States and in Britain, there have been numerous legislative attempts to limit young trans people’s access to gender-affirming medicine [16,17], despite evidence that such a law could increase mental health concerns such as suicide risk [13,17]. Compounding these health inequities, health care providers rarely receive sufficient education or training to provide care that is sensitive to, or tailored to the needs of, trans health care users [18,19]. The fear of nonaffirming or discriminatory and/or incompetent health care can contribute to delayed health care among trans persons and teaching their own health care providers, which has been shown to increase the odds of delaying needed medical care [20,21]. At the same time, this may explain much of the intracommunity, peer-to-peer sharing of health-related content via web-based platforms [22]. In this paper, we discuss why and how trans people’s health care inequities can be addressed by bolstering digital knowledge mobilization and community-engaged knowledge exchange.

Community-engaged research broadly refers to various processes and practices involving collaborative partnerships with communities and/or people who have been historically underrepresented within, and poorly served by, the academic knowledge production process (eg, trans communities). An aspiration of community-engaged research is to form a symbiotic relationship whereby communities, for instance, health care users and researchers, work together with a common goal such as improving access to care [23]. Knowledge mobilization and collaborative knowledge exchange are integral components of community-engaged research. Knowledge mobilization is an umbrella term referring to the collaborative flow of research knowledge including “knowledge synthesis, dissemination, transfer, exchange, and cocreation or coproduction by researchers and knowledge users” [24]. Knowledge mobilization activities are associated with a faster translation of research findings to achieve transformation of publicly held beliefs, changing provider practices, inciting policy change, and exchanging or coconstructing knowledge together with key stakeholders or knowledge users.

### Social Media, Knowledge Mobilization, and Exchange

The use of social media as a strategy for disseminating research findings beyond academic contexts has been previously established. Social media provides opportunities for knowledge to be publicly discussed and debated almost instantaneously by researchers, together with knowledge users such as educators, health care providers, health care users, and the general population. Croatian medical researchers have used Facebook to translate evidence-based medicine and high-quality health information to laypeople, health professionals, and journalists alike [25]. A 2019 scoping review identified a sharp rise in the use of Facebook, Twitter, and podcasts to translate knowledge between physicians and their trainees since 1996 [26]. In total, this scoping review revealed 12 different social media platforms used for knowledge mobilization. TikTok was not included in this list (likely because of the literature search parameters including studies published between 1990 and 2018). In another recent example, web-based discussions of hydroxychloroquine and remdesivir enabled public interdisciplinary medical discussions and the discrediting of false claims [27]. These constitute communicative spaces where diverging “viewpoints have the potential to circulate and be negotiated” [28]. TikTok has also been noted as a platform through which credible COVID-19 public health messaging circulates [29], for instance, the collective responsibility to keep communities healthy by practicing social distancing [30]. A literature review of young people’s use of digital technology highlighted that lesbian, gay, bisexual, trans, and intersex youth value storytelling about identity and concluded that there is a need to further establish how social media platforms influence young people’s peer-to-peer sharing of health information [31]. Trans people, in particular, use social media as a *vehicle of transformation* to artistically document their gender transitions through a process of coproduction and storytelling with other trans people [32], with trans women engaging in extensive community-based digital advocacy on the web through the hashtag *#GirlsLikeUs* [33].

Increasingly, knowledge exchange and coconstruction of knowledge occur on social media platforms. Before the COVID-19 pandemic, much of researchers’ community engagement work and knowledge mobilization occurred in person. However, due in part to widespread social distancing and lockdown policies intended to mitigate COVID-19 transmission, social media use has accelerated over the past year, as these technologies enable people to stay connected with family, friends, and community [34]. For instance, TikTok use exploded in 2020, with it being one of the most frequently downloaded smartphone apps [35]. Social media platforms now serve as replacement access points for communities and other social connections. For this reason, community-engaged researchers must continue to develop digital knowledge mobilization strategies to join communities in which they are organizing. At the present moment, trans communities are primarily connected on the web.

### TikTok: A Social Media Platform for Short, Creative Knowledge Mobilization Videos

A subsidiary of the Beijing technology company ByteDance, TikTok currently sits among the world’s most popular smartphone apps [35]. According to TikTok’s *Our Mission* section, “TikTok is the leading destination for short-form mobile video. Our mission is to inspire creativity and bring joy” [35]. The TikTok platform enables users to create, share, and otherwise digitally engage with short videos (<3 minutes), which are simply referred to by users as *tiktoks*. Approximately 800 million people are registered as TikTok account holders worldwide [36]. In 2020, the proportion of global internet users aged 16 to 68 years engaging with TikTok was estimated to be approximately 18% [37]. Although userbase demographics include significant diversity in terms of gender, ethno-racial, cultural, and other characteristics, approximately 42% of TikTok users are aged 18 to 24 years [29]. Importantly, people >30 years are increasingly joining TikTok, including health care providers, researchers, and educators [38].

Integrating music, text, and video together, tiktoks aim to be audiovisually entertaining, and the platform itself provides creators with several built-in tools useful for engaging in digital knowledge mobilization and the collaborative exchange of ideas. Not unlike other social media platforms, such as Twitter, the TikTok video comment section enables text-based responses. However, TikTok users are also provided with the option of creating a video response to text comments, which makes more nuanced and in-depth audio-video discussions possible. Using the video-response-to-comment option, a creator can produce an entirely new tiktok, which features the original comment in a speech balloon. The 2 additional popular TikTok video editing options are the *stitch* and the *duet*. Creating a stitch video involves responding to another user’s video by inserting—quite literally stitching—a segment of the original video to trigger the start of a new tiktok, establishing a public knowledge exchange between 2 users within the same short video. A duet serves a similar purpose; however, a duet typically amplifies and preserves the original video’s content while at the same time offering notes of endorsement or critiques toward the original information shared. In many cases with duets or stitches, the original tiktok creator explicitly requested other users to stitch or duet the video to answer a question or otherwise engage in a larger public discussion. Taken together, these video creation options bolster a creative, audio-video digital knowledge exchange environment through which TikTok users engage with one another’s videos. These in-house features of the TikTok app are especially conducive to community-engaged knowledge exchange, as they involve direct, audiovisual communication with other TikTok users.

Those who have experience with opening social media accounts on other platforms may appreciate that one of the first hurdles is gaining an audience (eg, subscribers or *followers*) with whom to exchange information surrounding particular topics. Contrasting with other social media apps built around individual subscribers, TikTok connects an international user base through a unique algorithm. Each TikTok user has a personalized *For You* page, which is fine-tuned by the TikTok algorithm, reflecting each user’s individual interests and engagement on the platform. The algorithm is sensitive to the user’s TikTok activity, including past videos commented on, liked, or shared, as well as the hashtags these videos apply. Not unlike other social media, it is primarily through all of these platform-based activities that a user’s *For You* page is curated by the algorithm [39]. The TikTok platform may ameliorate this barrier of finding an audience to engage with, given that when a video’s content resonates with other platform users, it may gain the attention of a large global audience in relatively little time as it is shown to other users [38]. TikTok videos shared, stitched, and duetted extensively by other users also tend to attract larger audiences, and the algorithm mirrors this activity by sending popular videos to be viewed by an increasing number of users, thereby compounding a trending video’s attention.

To demonstrate, Basch et al [40] sampled 100 popular, trending English-language tiktoks that applied the hashtag *#WearAMask* and compared these with 32 videos posted by the World Health Organization (WHO), which were also related to mask wearing. The metadata of each of the 132 sampled videos were collected and analyzed. Although the ratio of trending tiktoks to WHO videos was approximately 3:1, TikTok videos were viewed 500 million times versus the WHO’s 57 million views. The *#WearAMask* TikTok campaign received approximately 10 times the views, indicating *widespread reach* and TikTok’s transformative, community-engaged potential for communicating health information. Eghtesadi and Florea [41] also observed that TikTok is an important social media platform that could be used to share medical research with physicians and the general public. Useful for public knowledge exchange on topics of community concern, TikTok could help to achieve a wider discussion inclusive of individuals who may be otherwise inaccessible to academic researchers who use more traditional or in-person community engagement strategies.

The hashtags *#AcademicTikTok, #LearnOnTikTok*, and *#ProfessorsofTikTok* are just a few of the many examples demonstrating knowledge dissemination and educational content creation already occurring on the platform. These hashtags link researchers, educators, health care providers, and health care users together with diverse audiences around the world, creating innovative digital knowledge exchange. Some of the content uses humor to critically reflect on academia—for example, poking fun at the peer-review publication process—whereas other videos serve to share the results of a research paper and educate or even recruit study participants. For example, the popular science communicator Hank Green (@hankgreen1) boasts approximately 4.5 million followers. In the context of the digital trans TikTok community, the gender-affirming surgeon Dr Sidhbah Gallagher (@gendersurgeon), who shares information about gender-affirming surgeries, has approximately 165,000 followers. Peer-to-peer knowledge sharing is another form of digital knowledge exchange that often occurs on TikTok (and on other social media). Examples of peer-to-peer knowledge sharing happening on TikTok include instances of trans people sharing with other trans peers Dr Gallagher’s educational videos about gender-affirming surgery or tagging their peers in the comment section to notify them about the video. In another example of peer-to-peer sharing, the trans-identified physician Dr AJ Eckert (@drajeckert) creates tiktoks containing medical information about gender-affirming hormonal therapy intended for audiences of trans health care users and other health care providers.

Over time, knowledge mobilization has shifted from a positivist, linear, top-down translation of research findings to increasingly complex, iterative approaches that include knowledge coproduction in the context of its use [42]. Said differently, the former model is being replaced by knowledge activities that endorse research as a creative enterprise defined by human relationships, the salience of quality partnerships with health care users, and power-sharing techniques [43]. In fact, knowledge mobilization strategies have more reach and better engagement when involving collaboration or co-design with stakeholders, such as patients, thereby capitalizing on already established web-based community networks [44]. Accordingly, boundaries are increasingly porous between researchers and communities under study, as evidenced by the knowledge exchange happening on TikTok. The platform engenders not only the sharing of research findings by researchers but also collaborative knowledge exchange between all TikTok users through features such as the stitch and the duet. Such collaborative approaches harness co-design between researchers and knowledge users, in turn, minimizing power differentials while engaging underrepresented voices and valuing the legitimacy of lay, or community-level, knowledge [42]. In the following section, we further discuss why trans communities specifically present unique opportunities for collaborative and community-engaged knowledge mobilization via TikTok.

## Community-Engaged Knowledge Mobilization and Trans Peer-to-Peer Information Sharing

Trans people comprise approximately 0.5% to 1.3% of the general population [45]. Trans populations also confront significant barriers to health care, such as gender-affirming medicine, and experience discrimination within these settings [46]. Despite more public visibility of trans populations, trans-related stigma compounds social and health injustices [45]. Trans-related stigma occurs when ideologies, policies, and structural systems enforce a male/female binary that disadvantages trans people [47]. Trans-related stigma can occur at the systemic level through discriminatory policies and can be enacted at the interpersonal level through discrimination within individual interactions [48]. To demonstrate, the US Trans Survey found that 25% of trans-identified respondents encountered a problem with their health insurance provider denying claims related to gender-affirming medicine, with 33% experiencing at least one negative interaction with a health care provider such as verbal harassment or having to teach their own provider about appropriate care for trans people [20].

Disrupting trans-related stigma as well as sharing up-to-date empirical trans health knowledge about topics such as gender-affirming medicine are fundamental to improving health care access, health outcomes, and social conditions for this population. At the same time, social media platforms such as TikTok enable trans people to create alternative and trans-affirming knowledge that can counter stigmatizing beliefs. TikTok also provides a platform for health care providers and researchers who specialize in gender-affirming medicine to educate other clinicians and policy makers. Reflecting contemporary collaborative knowledge coproduction [42], trans communities are digitally well-connected and are active in peer-to-peer information sharing, support, and intracommunity education on the web [22,49,50]. This creates optimal conditions for community-engaged research and digital knowledge exchange between researchers, health care providers, and trans health care users. TikTok is especially popular with trans people, and *#TransTikTok #TransTok #NonbinaryTikTok and #GirlsLikeUs* are just a selection of hashtags that collate content and make peer-to-peer knowledge exchange possible. Then, through the power of the TikTok algorithm and individually curated *For You* pages, information relevant to this population is widely digitally disseminated via hashtags, duets, stitches, and the sharing and tagging of these videos between users.

However, it is important to stress the reality that trans people may seek out social media as a means of escaping a discriminatory world to connect with, and provide mutual support to, others who share a similar understanding of gender identity and expression. A 2021 scoping review found that social media and web-based communities were sources of both peer-based support and resilience for trans people, which in turn could buffer poor mental health outcomes experienced by this population [49]. Another 2021 scoping review identified *advocacy and education* as prominent themes of trans peer-to-peer information sharing and support [22]. Lupton [31] specifically recommends examining how TikTok and other social media platforms are used for health-related content creation and peer-to-peer sharing among young people. It is important to point out that peer-to-peer information sharing on trans health–related topics may also emerge in response to gaps in health professionals’ education and competency in this domain; thus, tiktoks could be shown to clinician learners in health professions education and training. This may explain, in part, why videos concerning gender-affirming medicine generate significant attention and knowledge exchange on TikTok. In the following section, the tiktoks created by KRM (@prof.kinnon; ~13,800 followers) are shared, and analytics data are presented.

## TikToking About Gender-Affirming Medicine: Digitally Mobilizing Knowledge

**Figure 1:**
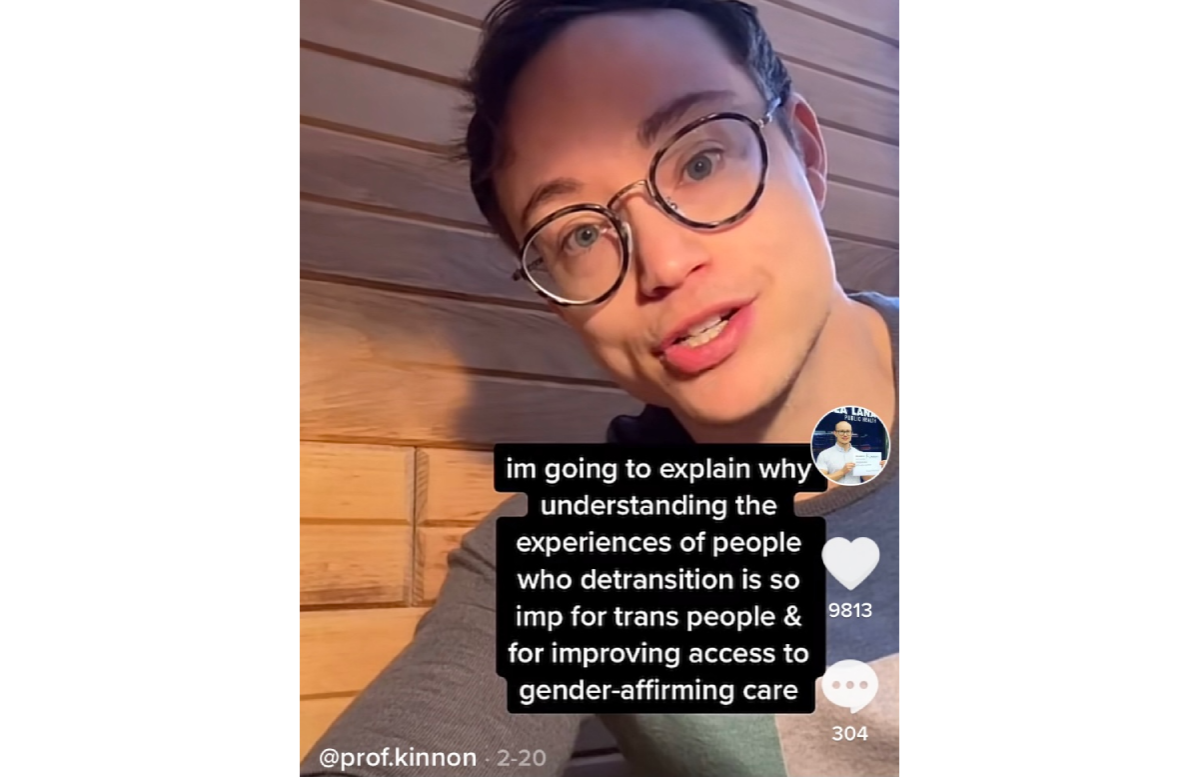
"Regret" and gender-affirming care.

We sampled 13 different tiktoks created by KRM and posted them between February 19, 2021, and March 4, 2021 (Multimedia Appendix 1). These tiktoks focus on gender-affirming medicine and primarily feature his own research and related studies by other trans health researchers. Each of these videos is <52 seconds in duration and covers academic research on topics of interest to health care providers and trans people seeking and receiving hormones and surgeries. Figure 1 shows one of these tiktoks about gender-affirming medicine eligibility assessments [51] that, to date, has received >70,000 views and 8746 positive endorsements (eg, likes or hearts). For our purposes, we simply present the number of views and shares for this compiled group of 13 tiktoks. At the time of writing (May 7, 2021), these videos were cumulatively viewed >378,301 times and shared >1313 times. Taken together, the creation of these short videos alongside much engagement from web-based trans communities via *#transtiktok* demonstrates the potential for innovative, digital knowledge mobilization and exchange on TikTok.

TikTok analytics data are included within each video, updated daily, and provided to every TikTok user with >1000 followers, enabling opportunities for researchers to monitor and measure the impact of their videos. These data provide researchers with important analytics metrics to track the impact and reach of their videos, such as the average view time, the total number of likes or hearts, and very basic viewer demographics (gender and country). Although counterintuitive, it is not uncommon for videos posted at an earlier date to gain traction with the algorithm at a later date, be pushed to users’ *For You* page, and subsequently go *viral*—a term used to denote a trending video. In such cases, a video has been liked, commented on, and/or shared by thousands of TikTok users, and the algorithm is sensitive to this activity, thus prompting the algorithm to showcase it on other users’ *For You* page [38]. The more that users watch and engage with individual tiktoks, the more the algorithm shows the video to other users with a history of engaging with videos of similar content. Thus, videos can gain TikTok users’ attention at discrete periods unrelated to the original time and date posted.

## Considerations for Researchers Planning to Use TikTok for Digital Knowledge Mobilization and Exchange

### Overview

Although researchers in past years primarily used mass media, including newspapers or radio, to communicate the latest scientific research study findings, the public’s newfound reliance on social media to access information about current events has increased. A survey commissioned by the Canadian Journalism Foundation found that 52% of Canadians and 48% of Americans aged ≥18 years use social media for news-related information [52]. In another example, survey data collected by Pew Research suggest that approximately 1 in 5 American adults access political news from social media, with 48% of those aged 18 to 29 years reporting social media as their main news source [53]. The demographics of these individuals who use social media as the main news source also tend to be “younger, are less likely to be white and have lower levels of education” [53]. Within the new digital age of disinformation and *fake news*, social media engagement is a contentious subject, particularly for academic researchers [54]. Social media platforms have clearly become indispensable tools for disseminating empirical knowledge widely and equitably.

TikTok is an innovative digital platform embedded with possibilities for transformative, collaborative knowledge mobilization and community-engaged knowledge exchange between researchers, underserved health care users, and their health care providers. As we have shown in discussing trans populations as an exemplar, TikTok presents unique opportunities for collaborative and community-engaged knowledge mobilization, and it may effectively promote critical dialog and information sharing to advance health care access for populations who experience poor health outcomes and barriers to care. However, there are several issues that researchers need to consider before launching their first tiktoks into the digital world. These can be summarized as follows: (1) TikTok analytic tools to measure knowledge mobilization and exchange, (2) population-specific safety concerns on social media, (3) the spread of disinformation and the need for legislation and regulation, (4) reaching those who have barriers to internet access, and (5) commercialization and intellectual property concerns.

Of relevance to those interested in using TikTok analytics to measure and evaluate the reach and impact of their videos, Basch et al [40] pointed out the challenge in distinguishing between the number of video views from viewers. Although TikTok provides users with viewer analytics data, it is not possible to identify when individual viewers watch a video multiple times or whether the videos are watched to completion. TikTok analytics do track the overall average watch time of each video; however, disaggregating these analytics data is not currently possible. These are platform-specific confounding factors that researchers must be aware of when seeking to measure and evaluate their digital knowledge mobilization activities on TikTok.

When leveraging TikTok for knowledge mobilization and exchange, there may be population-specific psychosocial developmental or safety considerations depending on the target stakeholder or equity-seeking group. For instance, youth aged <25 years comprise most of the TikTok users; however, social media use is positively correlated with depression, anxiety, and sleep disturbances in young people [55]. Some research cautions that youth engaging with health-related content on social media tend to compare their bodies with popular social media influencers [31] and that there are psychosocial risks, given the publicized evaluation metrics of likes, shares, and comment sections that allow users to openly criticize the video creator [34]. Similarly, young trans people use TikTok, and trans people of all ages regularly experience trans-related stigma on the web and in person. Trans people further report high rates of body image concerns and eating disorders, and a literature review published in 2016 concluded that body dissatisfaction is central to trans people’s psychosocial distress [56]. Moreover, Maly [39] noted that it is through the algorithm that these metrics contribute to the creation of a populist voice whereby followers, likes or hearts, and shares become *political facts*. However, a study of a Facebook group suicide prevention program with youth concluded that young people could be safely and effectively engaged on social media, even on psychologically sensitive topics such as suicide [57].

A possible solution to account for these population-specific safety risks is through the piloting of digital knowledge mobilization products, or knowledge exchanges, on other social media that offer private and anonymous engagement options. These alternative options may include private Facebook groups, Twitter, or Discord. Given TikTok’s powerful international algorithm and its stitch and duet video editing features, this platform may not always be the most accessible option for marginalized communities who are not public about their identities or who confront harassment on the web, such as trans people.

Although TikTok presents opportunities for collaborative knowledge exchange in the spirit of health equity and social justice, the app itself is not impervious to wider social discrimination. TikTok has admitted to *shadow-banning* or otherwise suppressing content created by people from minoritized groups, such as lesbian, gay, bisexual, trans, intersex, fat, disabled, and racialized people [58,59]. According to TikTok, creators from these groups may be more vulnerable to cyberbullying; therefore, the hashtags often used within these communities are rendered less discoverable by the algorithm [59]. In the wrong hands, TikTok’s stitch and duet film editing features could be used maliciously to misappropriate the original video’s message. Furthermore, the possibility of facing swapping or creating *deep-fake* videos has been noted [38]. Despite TikTok’s description of the functions of shadow-banning content, this measure may result in restrictions on the reach and impact of knowledge mobilization and exchange. Furthermore, there also exists disinformation and hateful content on the app, including expressions of transphobia [60]. Further analyses on the use of TikTok to share hate and right-wing propaganda has been demonstrated in the study by Weimann and Masri [60]. Minoritized populations have been misrepresented, and their experiences misinterpreted in knowledge creation and dissemination long before TikTok and other social media. This is a harsh reality that all researchers working with marginalized communities must be accustomed to thinking about and developing plans to account for these risks.

An obvious approach to disrupt antiscience, disinformation, or other related ideologies harmful to trans and equity-seeking populations is for academic researchers, health care providers, and educators to debunk these beliefs with research [54]. This could be done using TikTok’s stitch or duet video editing options to directly respond to disinformation with a new TikTok video or by downloading the original video and engaging critically with its content in the context of teaching and learning. Health professions educators should note that tiktoks can be shown to learners by linking the TikTok’s URL address or by downloading the video to a device such as a smartphone. Once stored on a smartphone, a TikTok video can be uploaded and shared on other social media platforms. Of note, each of these options risks amplifying harmful disinformation as the algorithm is sensitive to such activities; in response, the original TikTok video could be pushed to be viewed by more users of the TikTok app itself. Better legislation and regulation of TikTok are clearly required to prevent the spread of disinformation. In cases where researchers are aware that the content of their video may ignite significant controversy, it is possible to disable the duet and stitch options, the option for others to download the video, and turn off the comment section, thereby foreclosing the potential for tampering with the original, intended messages. Cautious researchers may consider disabling these features permanently, although in doing so, some of the collaborative knowledge co-design opportunities with others via stitch and duet would be limited.

Like other economically marginalized populations, trans people have varying access to smartphones and reliable internet connections because of individual socioeconomic and regional factors. Given the existing literature on the digital divide [61], it is plausible that trans adults living in rural regions with sparse access to the internet, those with limited resources to purchase a smartphone, and those who are older may face barriers to participating in these forms of digital knowledge exchange occurring on TikTok. The US Trans Survey of 27,715 respondents indicated that 15% of the sample were aged 45 to 65 years, with 3% aged >65 years [20]. Moreover, 30% of the participants who responded to an income question reported an annual household income <US $20,000 per year, with half of those reporting an annual household income < US $10,000 per year. Furthermore, 3% of the sample who responded to a technology question reported not having a cell phone, which accounted for 882 people [20]. Given that the US Trans Survey was administered in a fully web-based format, this prevalence may be even higher.

Some researchers may be hesitant to share study results using a commercial, for-profit app or may hold reservations because of intellectual property concerns. To this, we note that the TikTok platform includes an intellectual property and copyright policy which “protects original works of authorship (eg, music, videos, etc.)” and it pertains broadly to the expression of users’ ideas and the ways that videos or music are created rather than the ideas or facts contained within the videos or music. According to the TikTok policy, using copyrighted content may violate TikTok’s policies and can be removed [62]. Diligent researchers may consider consulting in-depth, professional intellectual property legal advice, as well as their own institutional affiliations and funding bodies, before communicating their study findings on TikTok. At the same time, community-engaged researchers who are planning to share study results on TikTok may want to proactively discuss with and elicit feedback from community members and/or health care providers at the early stages of their projects (before dissemination phases). In fact, TikTok could be used as a digital community engagement tool to solicit research topic ideas from health care users, and other stakeholders, before commencing a community-engaged research study.

Despite this myriad of considerations, TikTok may still be a potential digital mechanism for researchers to share knowledge with international audiences or to target specific knowledge user groups, such as health care providers or underserved health care users. We stress that, given the shifting landscape of social media as a primary information source, TikTok is a novel technology for collaborative digital knowledge mobilization and exchange with communities that are organized on the web and who engage in extensive peer-to-peer knowledge sharing, such as trans communities. Leveraging TikTok for community-engaged, digital knowledge mobilization and transformation is, therefore, an important strategy to achieve health equity for populations in need of better health care and social justice overall.

## Appendix

Multimedia Appendix 1 TikToks on trans health.

## Abbreviations

WHO: World Health Organization

## References

[1] Tsao S, Chen H, Tisseverasinghe T, Yang Y, Li L, Butt ZA (2021). What social media told us in the time of COVID-19: a scoping review. Lancet Digit Health.

[2] Littman L (2018). Rapid-onset gender dysphoria in adolescents and young adults: a study of parental reports. PLoS ONE.

[3] Restar A (2020). Terrific work from the legendary @ButNotTheCity. Twitter.

[4] Restar A (2019). The function and beauty of being a faculty is to speak truth and SCIENCE to power. Twitter.

[5] Ashley F (2020). ‘Rapid-Onset Gender Dysphoria’. Twitter.

[6] Restar AJ (2020). Methodological critique of Littman's (2018) parental-respondents accounts of "Rapid-Onset Gender Dysphoria". Arch Sex Behav.

[7] Ashley F (2020). A critical commentary on ‘rapid-onset gender dysphoria’. Sociol Rev.

[8] Literat I (2021). “Teachers Act Like We’re Robots”: TikTok as a window into youth experiences of online learning during COVID-19. AERA Open.

[9] Fiesler C, Beard N, Keegan B (2020). No robots, spiders, or scrapers: legal and ethical regulation of data collection methods in social media terms of service. Proc Int AAAI Conf Web Soc Media.

[10] Noble S (2018). Algorithms of Oppression: How Search Engines Reinforce Racism.

[11] Pasquale F (2015). The Black Box Society: The Secret Algorithms That Control Money and Information.

[12] Reisner SL, Radix A, Deutsch MB (2016). Integrated and gender-affirming transgender clinical care and research. J Acquir Immune Defic Syndr.

[13] Bauer GR, Scheim AI, Pyne J, Travers R, Hammond R (2015). Intervenable factors associated with suicide risk in transgender persons: a respondent driven sampling study in Ontario, Canada. BMC Public Health.

[14] Colizzi M, Costa R (2016). The effect of cross-sex hormonal treatment on gender dysphoria individuals' mental health: a systematic review. Neuropsychiatr Dis Treat.

[15] Scheim AI, Perez-Brumer AG, Bauer GR (2020). Gender-concordant identity documents and mental health among transgender adults in the USA: a cross-sectional study. Lancet Pub Health.

[16] Greensmith H, Moore M (2021). Health care for trans youth is under attack in UK — and it’s impacting the US. Truthout.

[17] Kidd KM, Sequeira GM, Paglisotti T, Katz-Wise SL, Kazmerski TM, Hillier A, Miller E, Dowshen N (2021). "This Could Mean Death for My Child": parent perspectives on laws banning gender-affirming care for transgender adolescents. J Adolesc Health.

[18] de Vries E, Kathard H, Müller A (2020). Debate: why should gender-affirming health care be included in health science curricula?. BMC Med Educ.

[19] MacKinnon KR, Ross LE, Gualdron DR, Ng SL (2020). Teaching health professionals how to tailor gender-affirming medicine protocols: a design thinking project. Perspect Med Educ.

[20] James S, Herman J, Rankin S, Keisling M, Mottet L, Anafi M (2016). The report of the 2015 U.S. transgender survey. National Center for Transgender Equality.

[21] Jaffee KD, Shires DA, Stroumsa D (2016). Discrimination and delayed health care among transgender women and men: implications for improving medical education and health care delivery. Med Care.

[22] Harner V (2021). Trans intracommunity support & knowledge sharing in the United States & Canada: A scoping literature review. Health Soc Care Community.

[23] Balls-Berry JE, Acosta-Pérez E (2017). The use of community engaged research principles to improve health: community academic partnerships for research. P R Health Sci J.

[24] (2021). Guidelines for effective knowledge mobilization. Social Sciences and Humanities Research Council.

[25] Puljak L (2016). Using social media for knowledge translation, promotion of evidence-based medicine and high-quality information on health. J Evid Based Med.

[26] Chan TM, Dzara K, Dimeo SP, Bhalerao A, Maggio LA (2020). Social media in knowledge translation and education for physicians and trainees: a scoping review. Perspect Med Educ.

[27] Gottlieb M, Dyer S (2020). Information and disinformation: social media in the COVID-19 crisis. Acad Emerg Med.

[28] Uldam J, Askanius T (2013). Online civic cultures: debating climate change activism on YouTube. I J Commun.

[29] Basch CH, Hillyer GC, Jaime C (2020). COVID-19 on TikTok: harnessing an emerging social media platform to convey important public health messages. Int J Adolesc Med Health.

[30] Mohamad S (2020). Creative production of 'COVID-19 Social Distancing' narratives on social media. Tijdschr Econ Soc Geogr.

[31] Lupton D (2021). Young people's use of digital health technologies in the global north: narrative review. J Med Internet Res.

[32] Raun T (2014). Video blogging as a vehicle of transformation: exploring the intersection between trans identity and information technology. Int J Cult Stud.

[33] Jackson SJ, Bailey M, Foucault Welles B (2017). #GirlsLikeUs: trans advocacy and community building online. New Media Society.

[34] Abidin C (2021). Mapping internet celebrity on tiktok: Exploring attention economies and visibility labours. Cult Sci J.

[35] (2021). Our mission. TikTok.

[36] Sehl K (2020). Everything brands need to know about TikTok in 2020. Hootsuite.

[37] (2021). Flagship report 2020. GlobalWebIndex.

[38] Anderson K (2020). Getting acquainted with social networks and apps: it is time to talk about TikTok. Library Hi Tech News.

[39] Maly I (2019). Algorithmic populism and algorithmic activism. Diggit Magazine.

[40] Basch CH, Fera J, Pierce I, Basch CE (2021). Promoting mask use on TikTok: descriptive, cross-sectional study. JMIR Public Health Surveill.

[41] Eghtesadi M, Florea A (2020). Facebook, Instagram, Reddit and TikTok: a proposal for health authorities to integrate popular social media platforms in contingency planning amid a global pandemic outbreak. Can J Public Health.

[42] Langley J, Wolstenholme D, Cooke J (2018). 'Collective making' as knowledge mobilisation: the contribution of participatory design in the co-creation of knowledge in healthcare. BMC Health Serv Res.

[43] Greenhalgh T, Jackson C, Shaw S, Janamian T (2016). Achieving Research Impact Through Co-creation in Community-Based Health Services: Literature Review and Case Study. Milbank Q.

[44] Elliott SA, Dyson MP, Wilkes GV, Zimmermann GL, Chambers CT, Wittmeier KD, Russell DJ, Scott SD, Thomson D, Hartling L (2020). Considerations for health researchers using social media for knowledge translation: multiple case study. J Med Internet Res.

[45] Winter S, Diamond M, Green J, Karasic D, Reed T, Whittle S, Wylie K (2016). Transgender people: health at the margins of society. Lancet.

[46] Abramovich A, de Oliveira C, Kiran T, Iwajomo T, Ross LE, Kurdyak P (2020). Assessment of health conditions and health service use among transgender patients in Canada. JAMA Netw Open.

[47] King WM, Hughto JM, Operario D (2020). Transgender stigma: a critical scoping review of definitions, domains, and measures used in empirical research. Soc Sci Med.

[48] Hughto JM, Reisner SL, Pachankis JE (2015). Transgender stigma and health: a critical review of stigma determinants, mechanisms, and interventions. Soc Sci Med.

[49] Kia H, MacKinnon K, Abramovich A, Bonato S (2021). Peer support as a protective factor against suicide in trans populations: a scoping review. Soc Sci Med.

[50] Andalibi N, Lacombe-Duncan A, Roosevelt L, Giniel C, Wojciechowski K (2021). LGBTQ persons? use of online spaces to navigate conception, pregnancy, and pregnancy loss: an intersectional approach. Transact Comput-Hum Interact (TOCHI).

[51] MacKinnon KR, Grace D, Ng SL, Sicchia SR, Ross LE (2020). “I don’t think they thought I was ready”: how pre-transition assessments create care inequities for trans people with complex mental health in Canada. Int J Ment Health.

[52] (2019). On behalf of the CJF, Maru/Matchbox interviewed a representative sample of 1,516 Canadians and 1,523 Americans. The Canadian Journalism Foundation.

[53] (2020). Americans who mainly get their news on social media are less engaged, less knowledgeable. Pew Research Centre.

[54] Pennycook G, Epstein Z, Mosleh M, Arechar AA, Eckles D, Rand DG (2021). Shifting attention to accuracy can reduce misinformation online. Nature.

[55] Primack BA, Escobar-Viera CG (2017). Social media as it interfaces with psychosocial development and mental illness in transitional age youth. Child Adolesc Psychiatr Clin N Am.

[56] Jones BA, Haycraft E, Murjan S, Arcelus J (2016). Body dissatisfaction and disordered eating in trans people: a systematic review of the literature. Int Rev Psychiatry.

[57] Robinson J, Bailey E, Hetrick S, Paix S, O'Donnell M, Cox G, Ftanou M, Skehan J (2017). Developing social media-based suicide prevention messages in partnership with young people: exploratory study. JMIR Ment Health.

[58] Rosenblatt K (2021). Months after TikTok apologized to Black creators, many say little has changed. NBC News.

[59] Botella E (2019). TikTok admits it suppressed videos by disabled, queer, and fate creators. Slate.

[60] Weimann G, Masri N (2020). Research Note: spreading hate on TikTok. Studies Conflict Terrorism.

[61] Chesser A, Burke A, Reyes J, Rohrberg T (2016). Navigating the digital divide: a systematic review of eHealth literacy in underserved populations in the United States. Inform Health Soc Care.

[62] (2021). Copyright policy. TikTok.

