# Supplementary material for: Examining TikTok’s Potential for Community-Engaged Digital Knowledge Mobilization With Equity-Seeking Groups
Source: J Med Internet Res. 2021 Dec 9;23(12):e30315. doi: 10.2196/30315 (PMC8704107; doi:10.2196/30315)
Supplement: Multimedia Appendix 1 [file jmir_v23i12e30315_app1.docx]

**Figure 1 – Trans health TikToks**

| **Number** | **Title** | **Number of views** | **Number of shares** |
| --- | --- | --- | --- |
| 1 | Detransition research | 11600 | 29 |
| 2 | “Regret” & gender-affirming care | 70100 | 78 |
| 3 | Stigma & detransition | 4465 | 21 |
| 4 | Trans health history | 7895 | 13 |
| 5 | Eligibility assessment | 16500 | 57 |
| 6 | HRT assessment | 1343 | 3 |
| 7 | Surgery assessments | 2372 | 8 |
| 8 | Informed consent model | 18200 | 3 |
| 9 | Surgical regret | 33600 | 102 |
| 10 | Core detrans | 2526 | 4 |
| 11 | Regret is unpredictable | 21100 | 36 |
| 12 | Trust science | 140700 | 724 |
| 13 | Bioethics | 47900 | 235 |
